# Effect of Nano-SiO_2_ on the Microstructure and Mechanical Properties of Concrete under High Temperature Conditions

**DOI:** 10.3390/ma15010166

**Published:** 2021-12-27

**Authors:** Piotr Brzozowski, Jarosław Strzałkowski, Piotr Rychtowski, Rafał Wróbel, Beata Tryba, Elżbieta Horszczaruk

**Affiliations:** 1 Department of Civil and Environmental Engineering, West Pomeranian University of Technology, Szczecin, al. Piastów 50a, 70-311 Szczecin, Poland; piotr.brzozowski@zut.edu.pl (P.B.); jaroslaw.strzalkowski@zut.edu.pl (J.S.); 2 Department of Chemical Technology and Engineering, West Pomeranian University of Technology, Szczecin, ul. Pułaskiego 10, 70-322 Szczecin, Poland; rp43903@zut.edu.pl (P.R.); rafal.wrobel@zut.edu.pl (R.W.); beata.tryba@zut.edu.pl (B.T.)

**Keywords:** concrete, nanosilica, mechanical properties, high temperature, microstructure, porosity

## Abstract

The aim of the research was to determine how the admixture of nanosilica affects the structure and mechanical performance of cement concrete exposed to high temperatures (200, 400, 600, and 800 °C). The structural tests were carried out on the cement paste and concrete using the methods of thermogravimetric analysis, mercury porosimetry, and scanning electron microscopy. The results show that despite the growth of the cement matrix’s total porosity with an increasing amount of nanosilica, the resistance to high temperature improves. Such behavior is the result of not only the thermal characteristics of nanosilica itself but also of the porosity structure in the cement matrix and using the effective method of dispersing the nanostructures in concrete. The nanosilica densifies the structure of the concrete, limiting the number of the pores with diameters from 0.3 to 300 μm, which leads to limitation of the microcracks, particularly in the coarse aggregate-cement matrix contact zone. This phenomenon, in turn, diminishes the cracking of the specimens containing nanosilica at high temperatures and improves the mechanical strength.

## 1. Introduction

Concrete as a structural material is widely used in construction due to its low cost, good mechanical performance, and relatively high resistance to water and fire. However, concrete is a quasi-brittle material, shows weak resistance to cracking and low tensile and flexural strength [1]. The development of modern engineering construction requires new properties of the materials used. They should ensure the proper load capacity, durable in various environments and demonstrate other specific and valuable characteristics. Modern cement concrete has not only high compressive strength but also other, never seen before properties, like antibacterial performance [2,3], self-cleaning ability [4,5,6], insulating properties, or self-repairing ability [7,8,9]. Obtaining such properties of concrete is possible, among others, by using various nanomaterials for cement and concrete production. The nanoparticles most often applied to the cement-based materials are nanometals (nanosilver, nanogold, nanocopper, nanoplatinum, nanopalladium), carbon nanostructures, like nanotubes or fullerenes, titanium dioxide (TiO_2_), silicon dioxide (SiO_2_), and zirconium dioxide [10,11].

In concrete technology, the nanosilica is used on the industrial scale as well as nanometric titanium dioxide TiO_2_ and new nanometric generation of superplasticizers (SP). Many investigations are carried out within the range of using carbon nanomaterials, mainly nanotubes and graphene, in the cement composites [12]. Most of these studies are conducted on cement paste and mortars due to the high costs of nanomaterials manufacturing.

One of the best-recognized nanomaterials used in concrete technology is silicon dioxide (nano-SiO_2_), commonly known as nanosilica. The nanosilica (NS) can replace in the future the silica fume (SF). NS reacts with lime (CH) during cement hydration and generates the production of hydrated calcium silicates (C–S–H) phase, improving the mechanical strength and durability of concrete [13,14,15]. Reduction of silica particles size to the nanoscale can significantly affect the cement hydration leading to substantial improvement of strength and compaction of cement matrix microstructure [16,17,18]. The studies described in [19,20] have confirmed that the superficial activity of NS in the presence of cement and water is significantly higher than that of SF and Portland cement itself. Consequently, the consumption of CH is much higher in the case of NS. Moreover, highly stiff C–S–H content is higher in the cement composites modified with NS than the composites containing SF [21]. NS added to the cement in the form of powder improves the 28-day compressive strength of concrete [22,23,24,25]. However, its effect on the compressive strength is most prominent in the first seven days of concrete curing [25,26]. NS increases the tightness of the cement matrix, thus improving the resistance of concrete to water penetration and carbonization [27].

Proper dispersing NS in the cement-based materials can accelerate the hydration of the cement paste and compact the paste’s microstructure [28]. NS can be obtained by chemical synthesis or grinding, e.g., the waste glass cullet. However, a significant problem in the industrial use of NS is still good dispersing in the cement matrix. The excess of nanoparticles can lead to their agglomeration due to the high surface energy, leading to the local increase of porosity and weakening mechanical performance [29,30]. NS in the powder form is usually added directly to the cement or together with mineral additions like ceramic powder or fly ash. As an industrial product, NS can occur in the form of liquid, as a water suspension containing an average of 10 to 50% of nano-SiO_2_, with the diameter of the particles from 40 to 140 nm [28].

It was found out in recent years that some nanomaterials can slow down the degradation of cement-based composites caused by high temperatures [31,32,33]. This ability is very significant for ensuring the better resistance of concrete under fire. The research described in [34,35] has shown that NS significantly improves the resistance of the cement composites to the high temperature since it gives higher residual compressive strength than the nonmodified specimens and reduces the width of the cracks. The above phenomena are attributed to a higher amount and better stability of the C–S–H phase in the presence of NS [36] and the effect of nanofilling the composite’s structure [37]. The effect of filling the micropores by NS and improved packing of the particles in the cement matrix causes the limitation of spalling during fire [35,38]. The “protective” features of NS at the high temperature are used for chemical modification of the other nanomaterials. The use of nanoshells of NS enables obtaining the specific performance of the cement composites at high temperatures [35,39,40].

The published results of testing the properties of the cement composites modified with NS at high temperatures were obtained for the cement pastes and mortars. There are few publications concerning the use of NS for the modification of concrete. However, investigations described in [41,42,43,44,45], carried out on the cement concrete at the high temperature, show that the concrete behavior in these circumstances can be different due to the presence of the coarse aggregate. The aggregate makes up about 70% of the volume of concrete, so it mainly decides on the mechanical performance of concrete at high temperatures. The contact zone coarse aggregate—cement matrix is also of great importance. The comparison of the obtained results is additionally difficult due to the various methods of the specimens heating and very significant differences in concrete compositions and the type of NS used.

The authors aimed to analyze the influence of NS admixture on the mechanical performance and microstructure of cement concrete at high temperatures. Particular attention has been paid to observing the changes in the structure of concrete in the coarse aggregate—cement paste interfacial transition zone (ITZ). Such an approach is innovative in the description of the durability of the concrete modified with NS at high temperatures. After analyzing the mortars modified with nanoparticles [39,40]. For this aim, they selected for investigation the concrete modified with 1, 3, and 5% of NS in relation to the cement mass, based on the literature overview and own previous research. The maximum grain size of the coarse aggregate (pebble gravel) was 8 mm. The aggregate, which does not cause spalling at high temperatures, was used [45]. The size of the specimens for the strength testing followed the standard. This size is rarely used in investigating concrete modified with nanomaterials because of the high cost.

In addition, the method of preparing the specimens for the mechanical tests was in accordance with the standard laboratory method. A unique process of applying the commercial NS suspension to the mixing water was used to ensure the best possible NS dispersion in the cement matrix. The method has been previously developed by one of the authors [28]. The technique is innovative and was not used so far for the cement concrete modified with NS. The concrete specimens were heated at 200, 400, 600, and 800 °C. The details of selecting the concrete components, their characteristics, and test methods are presented in the second section of the paper. The results of TG and MIP, connected with the changes in the coarse aggregate—cement matrix ITZ (images of surfaces obtained by SEM), were used to analyze the obtained results, presented in the third section of the article. The differences in cracking type in the ITZ were observed. These differences were then connected with the changes inside the matrix caused by a high temperature (TG analysis) and the changes in the structure and number of the pores caused by the NS admixture. The problem appeared to be so complex that it needs further detailed investigation with the use of, for instance, a nanoindentation technique within the ITZ or the atomic force microscopy.

## 2. Materials and Methods

### 2.1. Materials

The ordinary Portland cement CEM I 42.5 (Górażdże Cment S.A., Chorula, Poland) with a specific density of 3.11 g/cm^3^ was used for preparing the concrete and mortar specimens. The chemical composition of the cement is presented in Table 1. The aggregate used was river sand and natural gravel with a specific density of 2.65 g/cm^3^. The grain size distribution curves of the sand and gravel are presented in Table 2. Concrete was modified with commercial water dispersion of colloidal nanosilica Levasil NO8 (Nouryon Chemicals B.V, Amsterdam, Holland) with the nanosilica content of 50% of the product mass. The density of the dispersion was 1.40 g/cm^3^. The size of NS particles was from 20 nm to 160 nm. The tap water conforming to EN 1008 [46] was used as mixing water. Also used was polycarboxylate superplasticizer (SP) MasterGlenium ACE 430 (Master Builders Solutions, Myślenice, Poland).

### 2.2. Mixture Composition, Mixing Process and Forming Specimens

The constant water to cement ratio, w/c = 0.5, and contents of NS equal to 0, 1, 3, and 5% have been adopted in the designed cement pastes and concrete. The water contained in the dispersion was included in the mixing water. The paste specimens have been marked P0, P1, P3, and P5, according to the nanoadmixture content. The concrete specimens have been marked C0, C1, C3, and C5, according to the nanoadmixture content. The number corresponds to a percentage of the nanomaterial regarding the cement mass in the paste and concrete, respectively.

Before mixing the liquid components of the pastes with cement, the given amount of NS was added to the mixing water (Table 3). The obtained mixture was agitated using the mechanical stirrer with the rate of 2200 rpm and simultaneously by ultrasonication with 20 kHz. The mixing process took 5 min. Then, the SP was added, and mixing was continued by another 5 min. The prepared solution was added to the cement and mixed in the mortar mixer. After mixing, the paste specimens were formed in cubic molds with the size of 20 mm.

Preparation of the concrete mixes consisted in mixing the aggregate with cement in the laboratory mixer. Then, 2/3 of the mixing water was added to the mixed dry components, and the mechanical mixing was continued. The NS dispersion was mixed with the remaining 1/3 of mixing water and prepared in the same way as in the case of the cement paste, agitating by 5 min. Next, the given amount of SP (Table 4) was added, and the combined components were mixed mechanically and simultaneously ultrasonicated by another 5 min. The obtained solution was added to the other components of the concrete mix. Test specimens were formed in the steel molds of 40 mm × 40 mm × 160 mm and the cubic molds with a size of 50 mm. The concrete mix was placed in two batches and compacted using the vibration table.

The specimens were demolded after 24 h. Until testing, they were stored in water at the temperature of 20 ± 2 °C. Before structural and mechanical tests, the specimens were removed from the water and dried to the constant mass in the laboratory drier at temperature 105 °C.

### 2.3. Structural Characterization

Transmission electron microscopic analysis (TEM) was used for the NS examination. The FEI Tecnai G2 F20 S (Thermo Fisher Scientific, Waltham, MA, USA). Twin equipment was used with an accelerating voltage of 200 kV. The scanning electron microscopy (SEM) (FE-SEM Hitachi SU8020) equipped with the Energy Dispersive Spectroscopy (EDS) analyzer (EDSNSS312, Thermo Fisher Scientific, Waltham, MA, USA) was used for the determination of elemental content. The samples were excited with 20 keV electrons during elemental analysis. Before analysis, the samples were thoroughly ground and placed over a double-sided conductive carbon tape glued to the carbon SEM stub.

The particles size distribution was determined by Dynamic Light Scattering (DLS) with Zetasizer Nano ZS apparatus (Malvern Panalytical Ltd., Malvern, UK). The NS sample was 1000-fold diluted with deionized water until a transparent solution was obtained. The measurements were conducted immediately after dilution of the suspension and after 15 min of ultrasonication.

Temperature programmed desorption measurements were carried out in the synthetic airflow (99.999% purity, 30 mL/min) using a combined thermogravimetric (TG) and mass spectrometric (MS) analysis system consisting of the thermobalance (TG, Netzsch STA 449 C, Selb, Germany) and a mass spectrometer (QGA Gas Analysis System, Hiden Analytical Ltd., Warrington, England). A heating rate of 10 K/min with the final temperature of 1000 °C was applied. The mass spectrometer was used as a detector to determine water (m/z 18 signal) and carbon dioxide (m/z 44), both desorbing from samples during controlled heating in the thermobalance.

The analyzed materials X-ray diffraction (XRD) patterns were collected by an Empyrean PANalytical X-ray diffractometer (Malvern Panalytical Ltd., Malvern, UK) equipped with a copper tube (Cu Kα; λ = 0.154 nm). The measurement range in the 2θ range was 5–55° with a step size of 0.05. The PDF-4 + 2020 International Centre for Diffraction Data database and High Score Plus software (Malvern PANalytical Ltd., Malvern, UK) were used for phase determination.

SEM images of concrete specimens were taken using a Hitachi TM3000 electron microscope. After heating to 200, 400, 600, and 800 °C and at reference 20 °C, the cuboidal specimens with a few millimeter slices were cut from the concrete’s middle part. These cross-sections were then analyzed using an SEM microscope. The specimens were cut with a diamond saw. The surfaces of the concrete specimens were not additionally treated, which made it possible to observe the scratches and cracks resulting directly from the action of high temperature on the tested concrete.

Mercury intrusion porosimetry (MIP) was applied to assess the concrete’s pore characteristics. The central sections were cut out from the cubical specimens 50 mm × 50 mm × 50 mm. Then specimens sizes were about 7 mm × 7 mm × 2 mm. The test samples were selected to contain both coarse aggregate and the cement matrix. For each composite, two tests were conducted to verify the repeatability of the results. Before the tests, the specimens were dried to constant mass at 40 °C. The mercury surface tension was set to 0.48 N/m, and the contact angle was 140 degrees upon intrusion. The samples were put into measurement cells and filled with mercury in a low-pressure chamber. Subsequently, the cells were inserted into a pressure chamber and subjected to high pressures (up to ca. 413 MPa).

### 2.4. Mechanical Performance of Concrete

**Figure 1:**
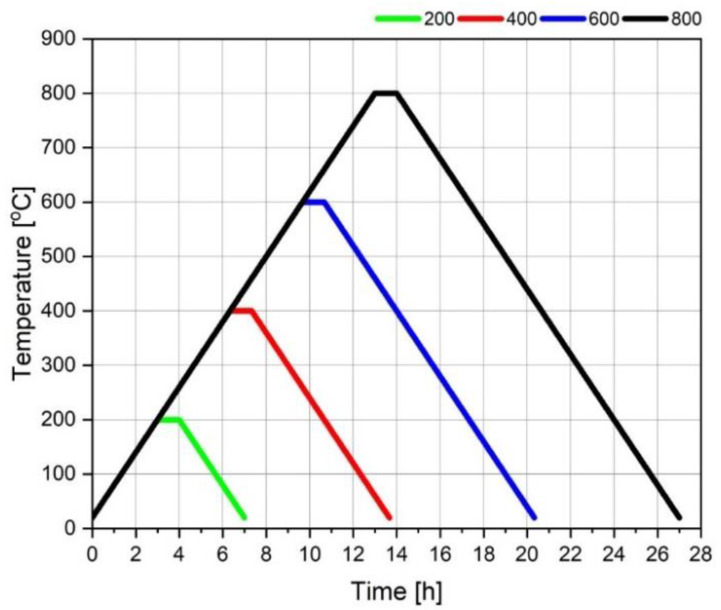
Heating and cooling regimes of the concrete specimens.

The tests of the mechanical properties of the concrete specimens were carried out at 20 °C and after heating at 200, 400, 600, and 800 °C. The specimens for heating were initially dried to the constant mass at 105 °C when they reached the age of 28 days. After weighing, the specimens were put into the medium-temperature oven (PP 140/85, LAC, Židlochovice, Czech Republic) and heated. The heating cycle at the given temperature has covered the heat to the proper temperature at the rate of 1 °C/min, maintaining this temperature for one hour, and cooling at the rate of 1 °C/min (Figure 1). After heating, the specimens were weighed, and their mechanical performance was tested.

The flexural strength was determined according to EN 12390-5 [47]. Three-point bending was used with a span of 120 mm. Because the biggest diameter of the used aggregate was 8 mm, the tests could be performed on the specimens with the size 40 mm × 40 mm × 160 mm. The results are presented as the average value from these three specimens.

The compressive strength was determined according to EN 12390-3 [48] on the cubic specimens with the size of 50 mm. The results are presented as the average value from these three specimens.

## 3. Results and Discussion

### 3.1. Characteristics of Nanosilica

**Figure 2:**
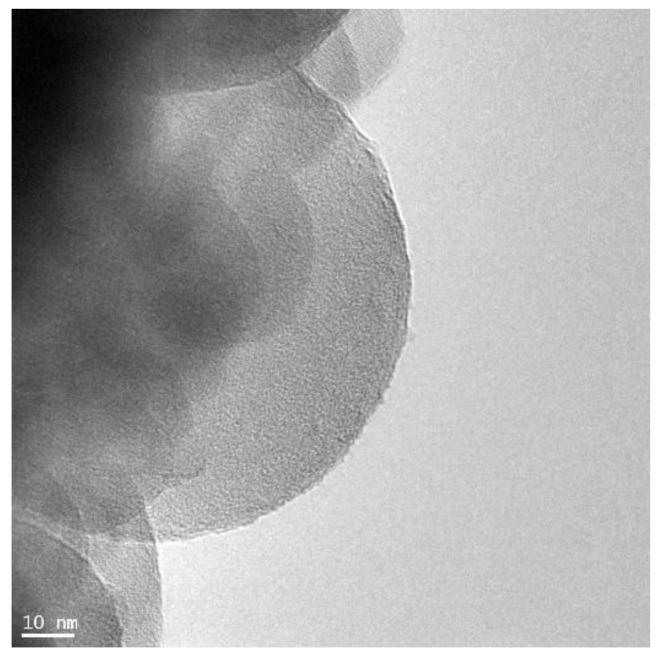
TEM images of nanosilica (NS).

**Figure 3:**
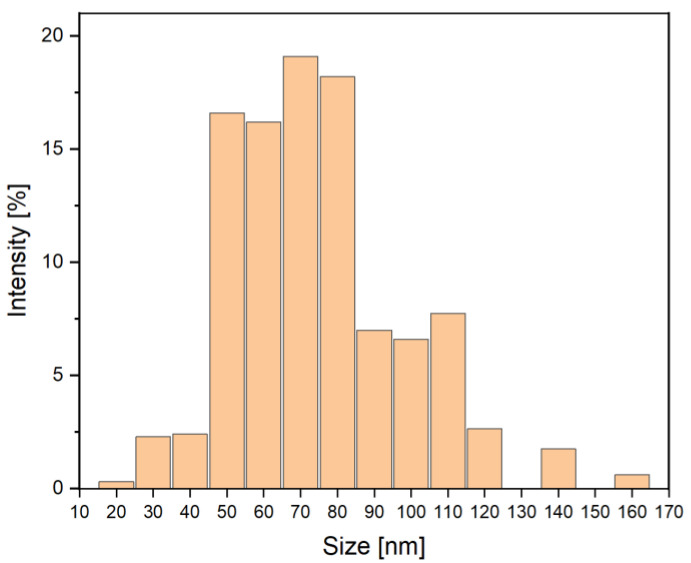
Distribution of NS particle sizes determined with the DLS method.

Figure 2 presents TEM images of nanosilica used for modification of cement paste and concrete. NS created stable dispersion in water. No significant difference was observed between the ultrasonicated samples and those without sonication. Three measurements were conducted; each of them was an average of 10–20 partial measurements. The number of partial measurements was a consequence of the given test sample stabilization. The sizes of NS particles were between 20 nm and 160 nm. Figure 3 presents a histogram with the average distribution of the particle sizes, calculated with the DLS method.

**Figure 4:**
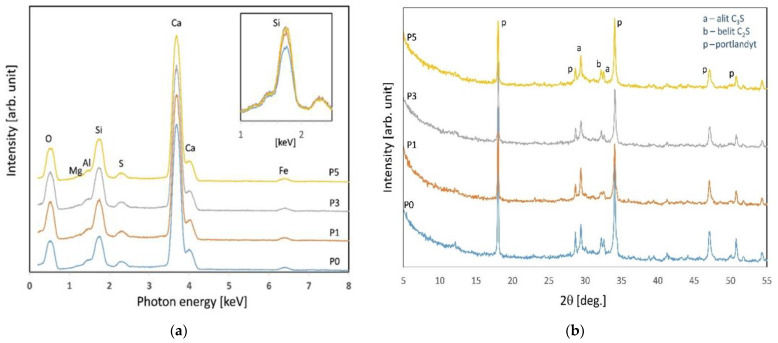
EDS spectra (**a**) and XRD spectra (**b**) of specimens after 28 days of curing.

Energy Dispersive X-ray Analysis gives elemental composition of cement pastes at 28 days (Figure 4). The visible increase in Si is confirmed by the modification of the cement paste with NS admixture. The collected diffractograms of investigated samples are presented in Figure 4 (right). For explanation of observed diffractograms the following phases were attributed: Ca_3_SiO_5_—alite (C_3_S), (ICDD 00-049-0442); Ca_2_SiO_4_—belite (C_2_S), (ICDD 00-033-0302); Portlandite, Ca(OH)_2_ (ICDD 00-044-1481).

All the samples exhibit similar phase composition to reference sample P0. The difference between the samples is the amount of amorphous phase SiO_2_. The amorphous phase is not visible on the diffractograms but affects the absolute intensity of reflections. Therefore, for the samples with the addition of colloidal SiO_2_ (P1; P3; P5), the absolute reflection’s intensity is lower than P0.

### 3.2. Thermogravimetric Analysis

**Figure 5:**
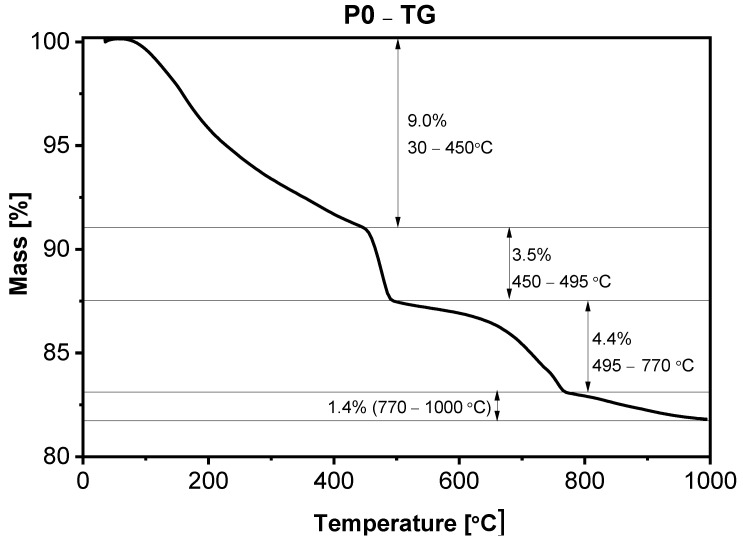
TGA of sample P0 after 28 days of curing.

**Figure 6:**
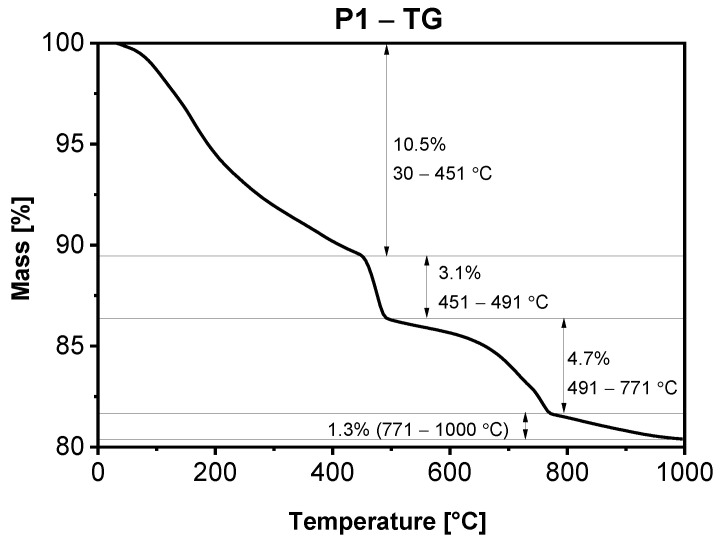
TGA of sample P1 after 28 days of curing.

**Figure 7:**
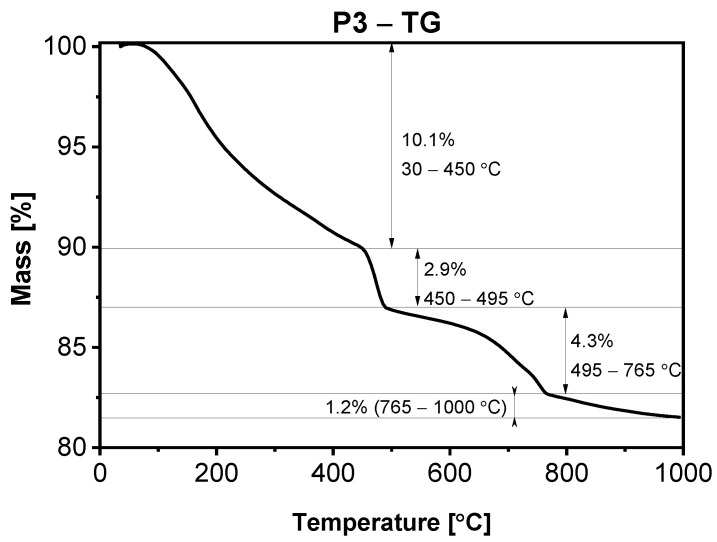
TGA of sample P3 after 28 days of curing.

**Figure 8:**
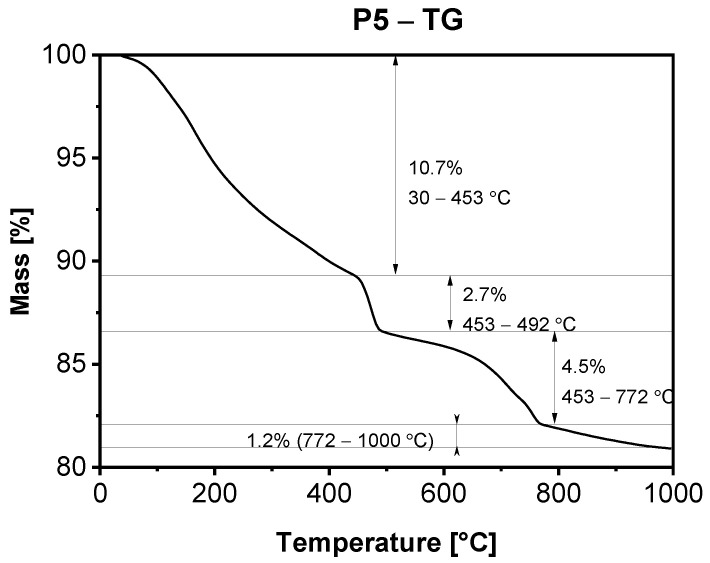
TGA of sample P5 after 28 days of curing.

**Figure 9:**
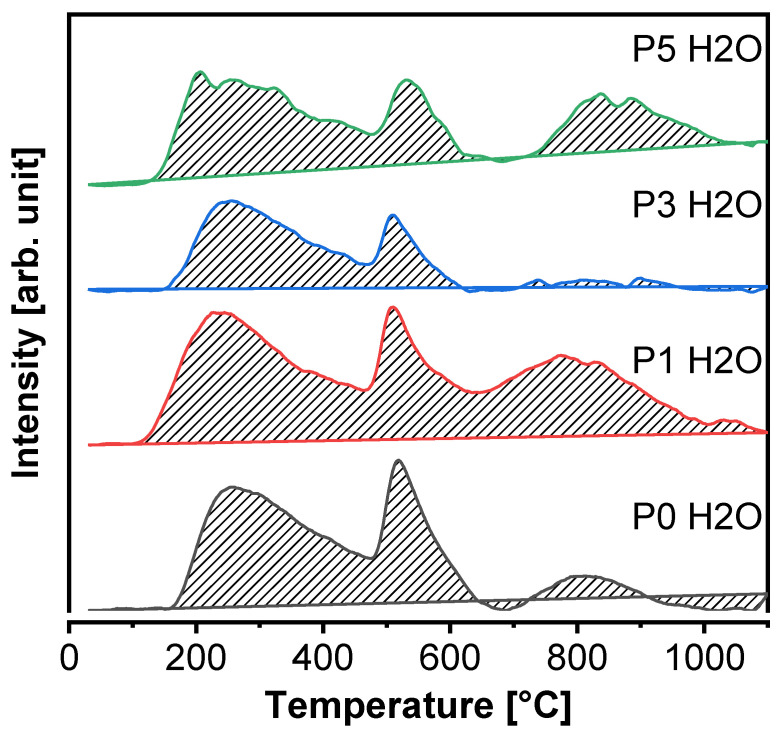
Plots of MS signal of water (m/z = 18) over time of paste samples after 28 days of curing.

**Figure 10:**
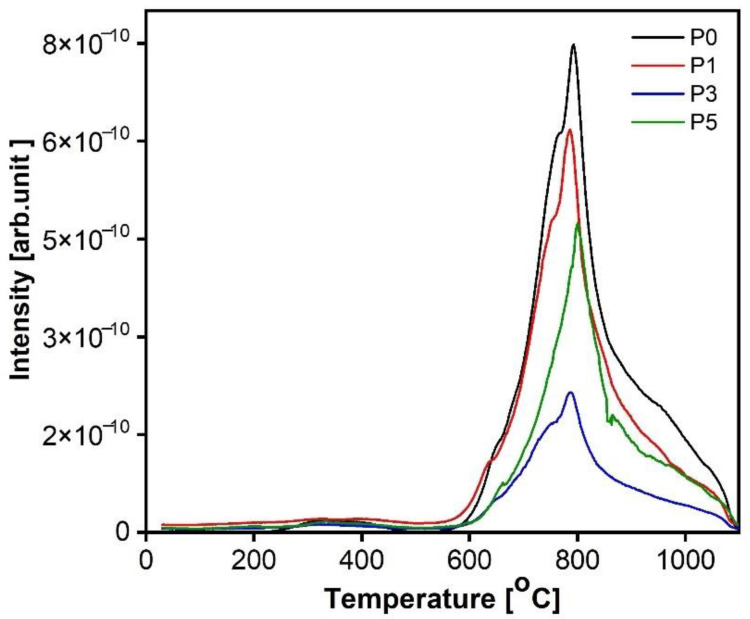
Plots of MS signal of carbon dioxide (m/z = 44) over time of paste samples after 28 days curing.

In Figure 5, Figure 6, Figure 7 and Figure 8, the results of thermogravimetric measurements are shown. The corresponding mass spectroscopy plots of H_2_O and CO_2_ within the time are shown in Figure 9 and Figure 10, respectively. All samples in the presented data indicate similar mass loss during thermal decomposition. Four significant steps of thermal decomposition can be distinguished. The first step occurs within the temperature range of around 30 to 450 °C and refers to the desorption of physically absorbed water. The second decomposition step also refers to water, but most likely chemically bonded to the sample’s surface, and is observed within the temperature range of around 450 to 490 °C. The third step appears within the range of 450 to 770 °C. This step is caused by the decomposition of the sample, leading to the release of water and carbon dioxide. The last step of thermal decomposition takes place at a temperature of around 765 to 1000 °C and leads to the smallest weight loss of the sample. Despite the fact that almost all TG curves underwent similar thermal decomposition in the context of weight loss, the amount of both water and carbon dioxide (Figure 9 and Figure 10) varies depending on sample type. The most visible differences occur in the last decomposition step (765–1000 °C), where samples P0 and P3 undergo decomposition with the release of only carbon dioxide, while samples P1 and P5 desorb both carbon dioxide and water particles. Taking into account the area under the MS signal curves, the conducted analysis indicates that the highest desorption of water occurred in case of P1 sample, while the highest amounts of carbon dioxide were observed during heating of sample P0. On the other hand, sample P3 desorbed the smallest amounts of both water and carbon dioxide. It can be concluded that the addition of 3% nano-silica to cement sample (P3) led to the biggest general improvement of thermal resistance which may be caused by creating stable chemical bonding of species present in the concrete sample.

### 3.3. Porosity Structure

**Figure 11:**
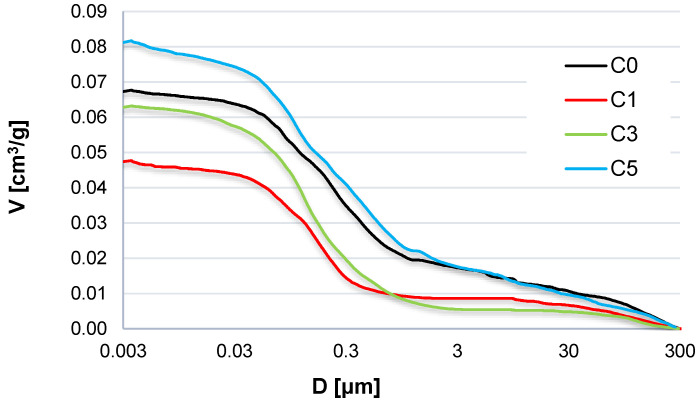
Cumulative porosity graphs of the tested concrete specimens (MIP).

The MIP method is widely used to describe porosity structure in porous materials, including cement-based composites [49]. It is used to analyze the problems regarding the durability of cement composites at high and low temperatures [26,50]. The results of the MIP tests are shown in Figure 11.

Modification of concrete with NS led to diminished porosity as compared to the reference concrete C0 in the case of two compositions, C1 and C3. Within the range from 30 to 300 μm, the specimens with NS have demonstrated slightly lower porosity than the reference concrete without the nanoadmixture. The admixture of NS significantly influences the porosity structure in the cement matrix, which is reflected in the average size of the pores and median of their volume in the specimens modified with NS (Table 5). Concerning the concrete specimens containing NS, the total porosity recorded by the mercury porosimeter increases significantly from 11.1% in concrete C1 to 17.2% in concrete C5. However, the high content of NS admixture can cause a total porosity increase, which was observed in specimens C5 with the total porosity higher than that of the specimens without NS (14.3%). The tests on the cement mortars modified with NS [49,50] confirm that nanosilica not necessarily causes an increase of cement matrix porosity but significantly affects the porosity structure.

### 3.4. Flexural Strength of Concretes before and after Heating

**Figure 12:**
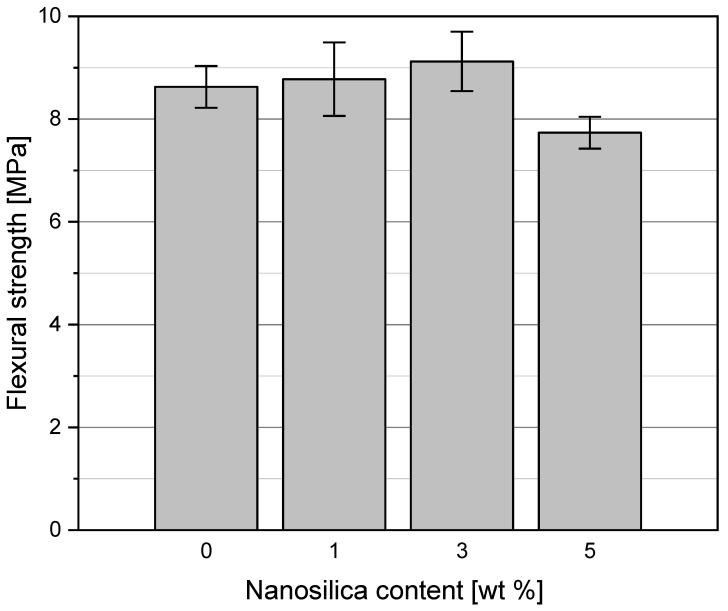
Flexural strength of concretes modified with NS after 28 days of curing (cured in water).

The flexural strength results of the concrete specimens after 28 days of curing in the water at 20 °C are presented in Figure 12. A slight improvement of the flexural strength of the concrete C1 and C3 in relation to the reference concrete C0 has been observed, by 1.8% and 5.7%, respectively. However, a decrease of the flexural strength of the concrete C5 in relation to concrete C0 by as much as 11% has been noted. Investigations described in [51,52,53] showed an average growth of the flexural strength by 5–15% was observed at NS contents from 2 to 3%, at various w/c ratios. The pozzolanic activity and the filling role of NS in the cement matrix both contribute to the increase of flexural strength [54]. However, a downfall of the flexural strength was observed with a further rise of NS content in the concrete [55], which is caused by the rapid growth of the cement matrix’s porosity and weak homogenization of NS in the composite.

**Figure 13:**
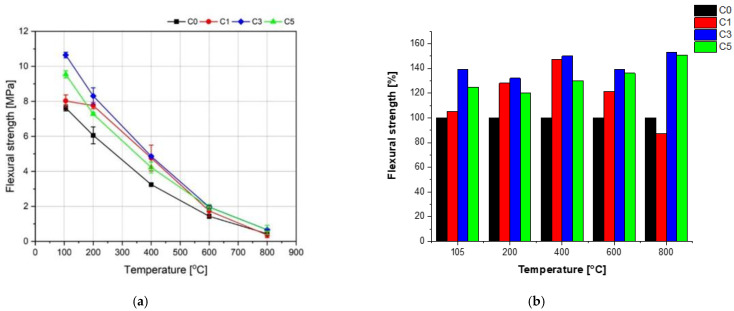
Influence of NS admixture on the flexural strength as a function of heating temperature (**a**) and analysis of flexural strength changes for the concrete specimens containing NS in relation to the concrete C0 without NS (**b**).

An influence of high temperature on the flexural strength of the tested concrete specimens was evaluated for the samples initially heated to the constant mass at 105°. The initial drying to the constant mass is used in most temperature tests of concrete [45]. The biggest downfall of the flexural strength has been observed for the concrete without NS (C0). A favorable effect of NS admixture on the strength at the high temperature is visible. Figure 13 (on the right) presents an analysis of the increase of the average flexural strength for the concrete specimens containing NS in relation to the concrete without NS (C0), which strength at the given temperature was accepted as 100% within the entire temperature range (Figure 13). It is shown that the NS admixture improved the flexural strength of the tested concrete specimens practically in the whole range of temperature. Only for the concrete C1, a decrease by 13% in relation to the reference concrete C0 at the temperature 800 °C has been obtained. The highest rise of the flexural strength with regard to the concrete C0 was observed for the concrete C3 containing 3% of NS. The growths of the flexural strength were 39, 32, 50, 39, and 53% at temperature 105, 200, 400, 600, and 800 °C, respectively.

### 3.5. Compressive Strength of Concretes before and after Heating

**Figure 14:**
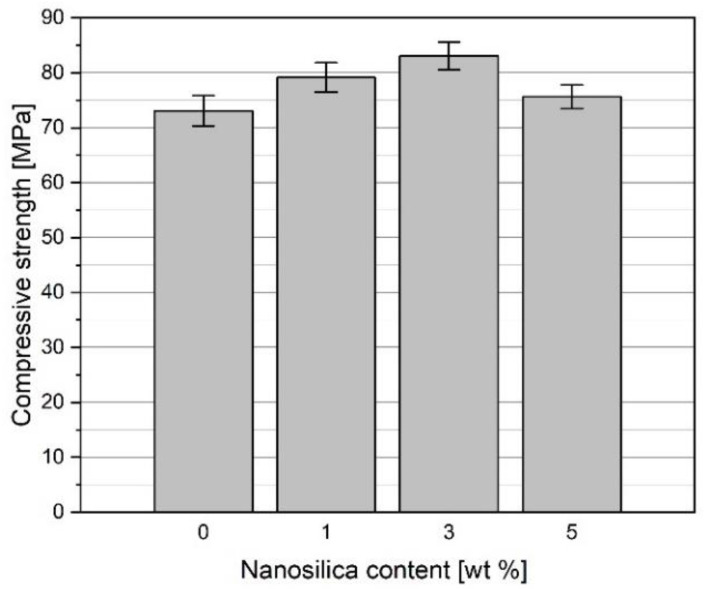
Compressive strength of concretes modified with NS after 28 days of curing (cured in water).

Figure 14 presents the compressive strength of the concrete specimens after 28 days of curing in water at 20 °C. The admixture of NS caused an increase in the compressive strength. The concrete specimens containing 1, 3, and 5% of NS showed growth of strength by 8.3, 13.7, and 3.5%, respectively, in relation to the concrete without NS (C0). The improved compressive strength of the concrete specimens C1 and C3 can be attributed to the lower total porosity of the specimens and the diminished number of the pores with sizes above 0.3 mm. The total porosity of the concrete C5 was higher, but the porosity structure was also changed, which enabled a slight increase of strength by 3.5% in relation to the concrete C0. The observed growth of the compressive strength is hard to compare with the results reported by other researchers due to the different sizes of particles and contents of NS, different w/c ratios, and other concrete additions. According to [56,57], the growths of the compressive strength after 28 days of curing at 2–3% content of NS were 10 to 19% compared to unmodified concrete. In the case of NS content higher than 5%, a decrease of strength was often observed. This worsening of the strength can be connected to the poor NS application technique, leading to a significant agglomeration of NS particles [35,49]. A deterioration of the concrete mix workability, even at the maximum amount of SP, was observed when preparing the specimens C5. The high water demand in the case of the significant NS volume has caused an increase in the total porosity of the concrete C5.

**Figure 15:**
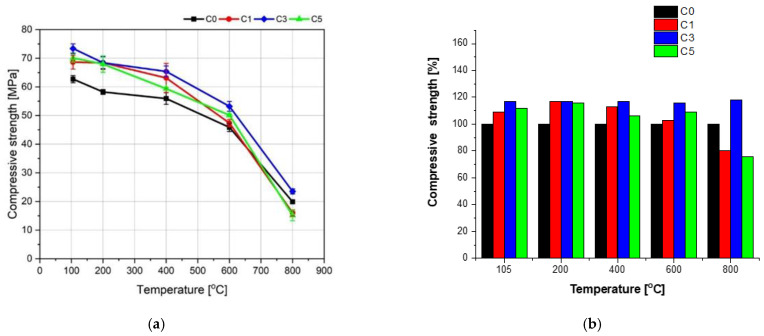
Influence of NS admixture on compressive strength as a function of heating temperature (**a**) and analysis of compressive strength changes for the concrete specimens containing NS in relation to the concrete C0 without NS (**b**).

Figure 15 presents the influence of NS admixture on the compressive strength of the tested concrete specimens as a function of heating temperature. A positive effect of NS admixture within the given temperature range was observed. However, the percentage increases of the strength of the concrete specimens containing NS about the concrete C0 were significantly smaller than those of the flexural strength and, except in one case, did not exceed 20%.

Similar to the flexural strength, the most significant rise of the compressive strength in relation to the concrete C0 was observed for the concrete C3 containing 3% of NS. The growths of the compressive strength were 13, 17, 17, 16, and 18% at temperatures 105, 200, 400, 600, and 800 °C, respectively.

### 3.6. SEM Analysis of the Coarse Aggregate—Cement Matrix Contact Zone in the Concrete Specimens Containing NS after Heating

**Figure 16:**
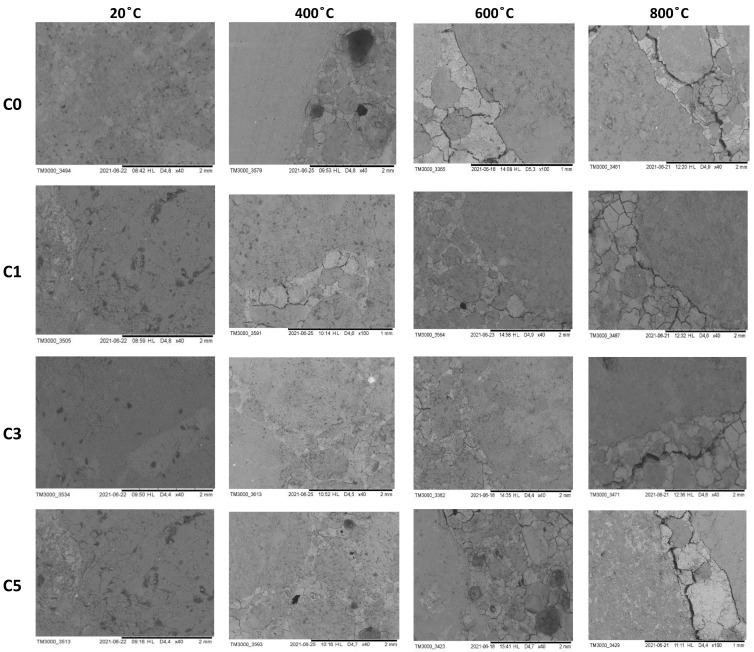
SEM images of the fractures of specimens with various NS contents after heating at a temperature from 400 to 800 °C.

Evaluation of the structural damages of the tested concrete was carried out based on the SEM observations of the heated concrete specimens. Since no cracks were observed at temperatures of 105 and 200 °C, these cases have been excluded from the analysis. Figure 16 presents exemplary SEM images of the fractures of specimens heated at a temperature from 400 to 800 °C.

The presence of NS limited the cracking in the cement matrix observed at 400 °C. Decomposition of the hydration products in these specimens is slower than in the unmodified concrete. The microstructural changes of the cement paste can be primarily attributed to the release of water and initiation of the hydrates’ decomposition. The TG analysis of the cement pastes (Figure 8) shows that decomposition of the hydration products at the temperature 400 to 600 °C is slower in the presence of NS than in the case of unmodified concrete. The cement matrix cracks were first observed on the border of the coarse grains at 600 °C for the concrete without NS. In addition, an increasing number of cracks in the cement matrix in the concrete specimens C0 and C5, demonstrating the highest porosity, were noted. The main cracks run through the pores weakening the cement matrix’s structure; the stress concentration generates the damage. After heating at 800 °C, all concrete specimens demonstrated the cracks on the border of the coarse aggregate grains. However, in the concrete C3, the cracks with significant width were mainly observed inside the cement matrix and not in the coarse aggregate—cement paste contact zone. The above phenomenon, together with the results of TG analysis and tight structure of the cement matrix, can explain the increased compressive strength of the concrete specimen C3 within the entire temperature range compared to the other specimens.

## 4. Conclusions

On the basis of the conducted investigations of the structure and mechanical performance of the concrete modified with NS at the high temperature, the following conclusions can be formulated:

1. The increase of the flexural strength of the concrete modified with NS compared to unmodified concrete at 20 °C is slight, and for the tested specimens was a maximum of 5.7%. The obtained results are hard to compare to those reported in the literature due to the various compositions of the concrete, including multiple sizes and contents of the applied NS. However, some influence of NS on the flexural strength of the cement concrete is demonstrated. For the concrete C5, a downfall of the flexural strength in reference to the concrete without NS was noted, mainly due to the increased total porosity of the concrete C5. A positive effect of NS admixture on the flexural strength of concrete has been observed within the considered temperature range.
2. The favorable effect of NS on the compressive strength of concrete at 20 °C was observed. The highest increase of the strength, by 13.7%, was noted for the concrete C3. It is hard to compare the obtained results with the investigations reported by other authors. The literature data show that the growths of the concrete strength in the presence of NS are smaller than for the cement pastes or mortars. According to the authors’ opinion, the NS content in the concrete C3 was so close to the optimum that despite the increasing porosity, the amount of water was sufficient to wet the NS particles and react to cement’s components to create more C–S–H phase with the higher stiffness. The above explanation has been indirectly confirmed by TG and MS investigations on the cement pastes.
3. The presence of NS in the concrete improved the compressive strength at a high temperature. The effect was less significant than the flexural strength; the increase averaged about 20% within the temperature range from 105 to 600 °C. What is essential, at 800 °C, a favorable effect of NS on maintaining the compressive strength was also observed (an increase by 18% in relation to the concrete C0 in the case of the concrete C3). It has been observed that in specimens C3 at 800 °C, the main crack occurred in the cement matrix and not on the coarse aggregate border. The above phenomenon could be the reason for the higher residual strength at this temperature, which, in turn, could be evidence of strengthening the coarse aggregate—cement matrix contact zone. ITZ is a crucial place in ordinary concrete from the point of view of durability at a high temperature. The described phenomenon seems to be very interesting and needs further investigation.
4. The improvement of the mechanical performance of the concrete containing NS at a temperature up to 600 °C is caused by compaction of the structure and the creation of the C–S–H phase with high stiffness (resulting from the longer silicate chain C–S–H). This explanation was confirmed by TG and MS investigations. The above assumptions require further testing with the use, for instance, the nanoindentation technique or atomic force microscopy.
5. In general, a positive influence of NS admixture on the concrete mechanical performance at a high temperature was demonstrated. It has been confirmed that there is an optimum content of NS enabling the creation of the best structure at the given concrete composition, which, in turn, allows the improved concrete resistance to high temperature. For the tested concrete compositions, the optimum content of NS was 3% of the cement mass.
6. The method of dispersing NS in the mixing water appeared ineffective in the case of the concrete C5. The superplasticizer content was too low to evenly disperse the large amount of NS in the cement matrix, which led to the increased porosity and worse mechanical performance of the concrete.

## Figures and Tables

**Table 1:** Chemical composition of Portland cement [wt.%].

CaO	SiO_2_	Fe_2_O_3_	SO_3_	Al_2_O_3_	MgO	K_2_O	Cl^−^	Na_2_O
63.77	21.07	4.12	3.2	3.11	0.58	0.37	0.071	0.05

**Table 2:** Chemical compositions of the aggregate [wt.%].

Aggregate	Sieve [mm]/Remains on the Sieve [%]								
	0	0.125	0.25	0.5	1	2	4	8	16
Sand	5.7	0.3	37.7	46.5	6.7	2.6	0.5	0	0
Gravel	0.3	0.9	0.9	1.9	3.2	35.1	55.4	2.3	0

**Table 3:** Proportions of the cement pastes.

Designation	Cement (C)	Water	Nanosilica (NS)	Superplasticizer (SP)
P0	1.0	0.5	0.0	0.006
P1	1.0	0.5	0.01	0.010
P3	1.0	0.5	0.03	0.018
P5	1.0	0.5	0.05	0.026

**Table 4:** Mix compositions of concrete [kg/m^3^].

Designation	Sand 0–2 mm	Gravel 2–8 mm	Cement	Water	NS	NS/C	SP	SP/C
C0	950	950	340	150	0.00	0.0%	2.04	0.6%
C1	950	950	340	147	6.8	1.0%	3.40	1.0%
C3	950	950	340	140	20.4	3.0%	6.12	1.8%
C5	950	950	340	133	34.0	5.0%	8.84	2.6%

**Table 5:** The basic properties of the tested concrete obtained by MIP.

Specimen Type	C0	C1	C3	C5
Total surface area [m^2^/g]	3.120	3.369	3.601	5.310
Pore tortuosity [–]	2.077	2.149	2.108	1.957
Permeability [nm^2^]	0.003	0.001	0.002	0.003
Total porosity [%]	14.152	11.112	13.819	17.241
Average pore volume [cm^3^/g]	0.054	0.040	0.050	0.068
Average specific surface area of pores [m^2^/g]	0.612	0.866	1.003	1.060
Median pore volume [cm^3^/g]	0.034	0.024	0.032	0.041
Median specific surface area [m^2^/g]	1.560	1.685	1.801	2.655

## Data Availability

The data presented in this study are available on request from the corresponding author.
